# Language-supported labor ward visits for pregnant migrant women: Staff experiences in a Swedish hospital

**DOI:** 10.18332/ejm/149519

**Published:** 2022-07-18

**Authors:** Anna Akselsson, Lisa Cabander, Steinunn Thorarinsdottir, Rhonda Small, Elin Ternström

**Affiliations:** 1 Department of Women's and Children's Health, Karolinska Institutet, Stockholm, Sweden; 2 Department of Health Promoting Science, Sophiahemmet University, Stockholm, Sweden; 3 Judith Lumley Centre, School of Nursing and Midwifery, La Trobe University, Melbourne, Australia; 4 School of Education, Health and Social Sciences, Dalarna University, Falun, Sweden

**Keywords:** pregnancy, birth, migrant women, language-assisted support, healthcare provider experiences

## Abstract

**INTRODUCTION:**

The aim of this study was to explore midwives’ and assistant nurses’ experiences of providing extra support to non-Swedish-speaking migrants by offering individual language-supported visits to the labor ward during pregnancy.

**METHODS:**

Semi-structured interviews were conducted with six guides, midwives or assistant nurses, working in the INFÖR (Individuell förlossningsförberedelse) project at Södertälje hospital in Sweden. INFOR includes a two-hour individual language-supported visit at the labor ward, for non-Swedish speaking pregnant women and their partners. An inductive thematic analysis was conducted.

**RESULTS:**

The guides described INFOR as being a bridge and creating safety, achieved by meeting with women and providing practical information. The guides felt that they fulfilled an important purpose, they were dedicated and adapted to the women’s individual needs. Providing extra language-assisted support to migrant pregnant women was developing and enriching, but the guides highlighted some barriers. Communicating via an interpreter was a challenge and the women were in need of more and extended meetings. The guides wished that INFOR could become a standard part of antenatal care, but the model needs to be further developed, and a better system for recruitment must be introduced.

**CONCLUSIONS:**

The guides experienced that the INFOR model is valuable in creating safety to pregnant migrant women before birth. The model is appreciated by the expectant couples, midwives and assistant nurses, and could be implemented as standard care. However, it is important to adapt the visits to the women’s and their families’ needs and goals, and structure needs to be developed before implementation.

## INTRODUCTION

Compared to non-migrant women, women migrating to industrialized countries have a 50% higher risk for perinatal mortality, preterm birth and having a baby with low birthweight according to a systematic review including 12 European countries^1^. Further, results from a meta-analysis including 42 million women, show that women who migrated to Western Europe have a doubled risk of dying when compared to non-migrant women^2^. Women migrating to western industrialized countries are also more likely to receive inadequate prenatal care and have fewer visits to planned antenatal care compared to non-migrant women^3-5^.

The definition of migrants used in this study is based on the definition by the International Organization of Migration (2019) defining migrants as: *‘any persons who have moved across an international border from their habitual place of residence regardless of their legal status, cause, length of stay, and whether the movement was voluntary'*^6^. Migrant and non-migrant women have similar desires and expectations of maternity care, i.e. feeling safe, receiving high quality care and being offered individualized care with adequate information and support^7^. However, migrant women are less positive about their care and have poorer self-rated health compared to non-migrant populations^7,8^. Poor communication and lack of connection with healthcare professionals are common experiences among migrant women during pregnancy and childbirth and are important areas for improvement^9^.

Cultural differences, different educational levels, different expectations of support, linguistic barriers and lack of knowledge about how the healthcare system works are some of the challenges reported by maternity service providers when caring for foreign-born women^10,11^. According to a Swedish study, when caring for migrant women lacking fluency in Swedish, midwives found that communication could work well despite language difficulties, if they showed real interest in the woman and her issues, so mutual understanding could be reached^12^. Creating a trusting relationship in this way increased understanding and led to a more meaningful connection. Healthcare inequalities among migrants are suggested by midwives to arise due to lack of time, language barriers, cultural clashes and limited trust between the patient and caregiver^13^. To improve healthcare for migrants, WHO recommends a person- centered model of care and encourages interventions that empower and increase migrant women’s and their families’ health literacy and knowledge of the healthcare system^14^. One such intervention, based on a leaflet and an app with information in different languages, has shown positive results according to the participating midwives, who felt that it was advantageous for the women to have information in their own languages also at home^15^.

Sweden has gradually evolved to become a multicultural society and at the end of 2019, 25.5% of Sweden’s population were of foreign background^16^. In Sweden antenatal care is free of charge and community-based midwives are responsible for the care of all uncomplicated pregnancies^17^. Birth care occurs with different midwives in hospitals; continuity of midwifery care is rare and while homebirth remains a possibility for women it is not offered as a standard option for care. For newly arrived families, midwives in maternity care might be the first contact they have with healthcare in Sweden. According to Swedish law, a person who does not speak or understand the Swedish language has the right to an interpreter^18^. In Södertälje municipality in Sweden, more than half of the inhabitants are of foreign background^19^. Based on research and their own experiences caring for migrant women, the healthcare staff identified that migrant women were a vulnerable group with higher risks for complications and greater need for support during pregnancy. The individual childbirth preparation project, INFOR *(inför* in Swedish, before in English), was started by midwives and assistant nurses to guide migrant non-Swedish speaking pregnant women through the healthcare system, establish personal contact with staff on the labor ward and thereby increase the women’s feelings of safety before, during and after childbirth.

This study is part of a larger evaluation of the INFOR-project. The aim of this study was to explore INFOR-guides’ experiences of providing this extra support to non-Swedish- speaking migrants by offering individual language-supported visits to the labor ward during pregnancy.

## METHODS

### Design and setting

The design of this study was qualitative with an inductive approach and a descriptive design^20^. The study was performed at Södertälje hospital in Sweden, a hospital with 2250 births a year. INFOR was first developed in 2016 by a group of experienced labor ward midwives and assistant nurses to improve care for non-Swedish-speaking, expectant parents to provide a better foundation for a safe birth and supportive care when women come to hospital to give birth.

INFOR is an ongoing project financed by the Södertälje hospital, offering non-Swedish-speaking pregnant migrants unfamiliar with the maternity care system in Sweden, an individual, language-assisted, two-hour birth preparation visit to the labor ward with a guide (a midwife or assistant nurse). The guide and the woman, and most times also the partner, meet to talk, together with an interpreter, and go on a tour of the labor and postpartum wards. The purpose of the visit is to give the expectant parents information about the healthcare system, what to expect giving birth and other practical details to help them prepare for their birth and the postpartum period. Women usually receive information about INFOR from their community-based antenatal care midwife at a regular visit during pregnancy, but they can also contact INFOR themselves. According to the guides, information about the project has also spread between women by word of mouth. The INFOR-project uses the same interpreter service as the antenatal care midwives, making it possible for the participants to request an interpreter they have met before. At the time of this study, 85 women had met a guide in INFOR (approximately two years from the start).

### Recruitment and participants

Guides who were active in INFOR were recruited to the study by email. Of the eight guides involved at the time of the study, six chose to participate. The six participating guides consisted of four midwives and two assistant nurses who had worked in their professions from 3 to 30 years and in maternity care between 1 and 28 years. Three of the participants had been guides in INFOR since it started three years previously, and the other three participants for between four months and two years. In Sweden, midwifery education involves four and a half years at university level (nursing studies in the first three years, followed by 18 months of midwifery education) and assistant nurses have undertaken three years of vocational education in nursing care at the equivalent of upper secondary level. In Sweden, assistant nurses are regulated healthcare professionals who often work in pairs with the midwives on labor ward caring for women giving birth.

### Data collection

Six semi-structured interviews were conducted between 31 January and 15 February 2019, with the support of an interview guide developed for the study by the authors. With guidance from author ET, who is a midwife with experience in qualitative studies, the authors ST and LC, who were both midwifery students at the time, conducted three interviews each. The interviews were performed at a time and place that suited the participants. All participants chose to meet in connection with a work shift on the labor ward. The interview guide started with some background questions about the specific guide, and continued with questions about: 1) the guides’ experiences of working with INFOR, 2) the guides’ views on the INFOR-participants experiences, and 3) challenges for the guides and potential development of the INFOR model. Follow-up questions such as: ‘Can you tell us more about that?’ or ‘What do you mean by that?’ were asked when needed. The interviews were recorded using the authors’ mobile phones, lasted between 19 and 47 minutes (average: 33 min) and were transcribed verbatim.

### Data analysis

The transcripts were analyzed with inductive reflexive thematic analysis as described by Braun and Clarke^21,22^. The themes were identified at a semantic level, i.e. patterns in the transcripts are shown through organization and description of the data. This process includes six phases and the authors moved back and forth between the phases during analysis. Familiarization with the data began during transcription and to get acquainted with the data, the transcripts were read repeatedly by ST, LC, ET and AA who is a midwife with experience in qualitative studies. Initial codes were generated through the entire data set, and the authors coded all interviews independently. The codes were then discussed, analyzed and combined into meaningful groups and patterns. Potential themes were identified, and to get a better overview a mind map of these themes was drawn. After that, the codes were read through again and discussed with regard to the themes in which they were placed. This process eventually led to one overarching theme, three themes and seven subthemes. At the end of this phase, all codes had been allocated to existing themes and subthemes. All five authors, including RS, who has extensive experience in both qualitative methods and research with migrant women, discussed whether and how the themes responded to the purpose of the study and ensured that the codes did not belong in any other theme than the chosen one.

### Ethical considerations

This study was approved by the Research Ethics committee in Stockholm, Sweden. All participants in the study received both oral and written information about the study and signed a consent form. They were also assured that the data collected would be treated confidentially and received information that participation in the study was voluntary and that they could choose to withdraw participation at any time, without stating the reason.

**Table 1:** Example of analysis process

Quote	Code	Subtheme	Theme
‘… to make the women feel safer in this way and perhaps reduce the risks. And in order for them to achieve the same foundation as Swedish speaking women, you need to do something extra so that they have the chance to learn more and feel more secure …’	Reduce the risks, same conditions as Swedish-born, create safety	Being genuinely engaged with each participant	Creating safety for the women and their families

## RESULTS

**Figure 1:**
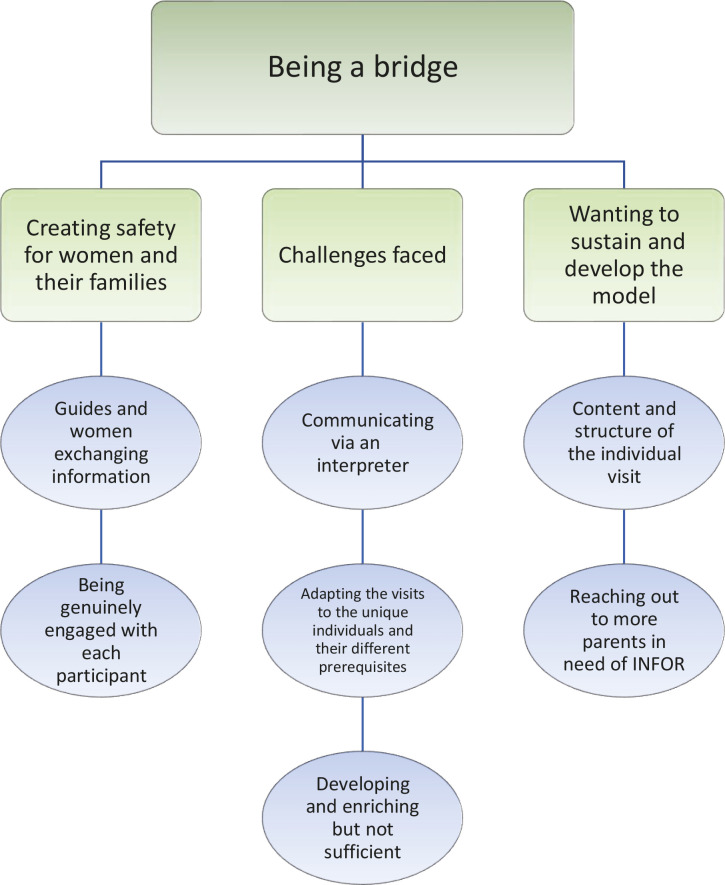
Results with themes and subthemes

After interviewing the INFOR-guides about their experiences of providing extra support to non-Swedish-speaking migrants, one overarching theme *Being a bridge* comprised three themes and seven subthemes, shown in Figure 1.

*Being a bridge* signifies how the guides saw their key role in INFOR as bridging gaps in the quality of care for participants in INFOR by informing women about Swedish maternity care and what they could expect at birth, as well as listening to and understanding women’s own needs and expectations, with the help of an interpreter. This was primarily accomplished on a personal level, by showing interest in each woman and adjusting the focus of the visit accordingly, but also on an organizational level bridging commonly experienced communication and information gaps. The three themes were: *Creating safety for women and their families, Challenges faced and Wanting to sustain and develop the model.*

### Creating safety for women and their families

The guides had a personal interest in improving care and building a bridge between migrants and the Swedish healthcare system. The guides agreed that there was no typical visit, however every visit had a guideline and an agenda. Two subthemes capture how the guides described key aspects of creating safety for women and their families and developing confidence in the care they would receive during labor and birth: *Guides and women exchanging information and Being genuinely engaged with each participant.*

*Guides and women exchanging information*

The guides often started the visit by explaining its purpose to the expectant parents. To assist with the structure, the guides often used a written guide for the visit. This included questions covering the families’ social circumstances, cultural background, past childbirth experiences and any of their concerns. The dialogue consisted of an exchange of information where the woman and her companion conveyed their experiences and thoughts and the guide shared information concerning childbirth and the care they would receive.

Practical issues such as costs, how and when to get to the hospital and if you should bring your own food were commonly brought up by the expectant parents during a visit. The guides felt that the tour of the maternity and postpartum ward at the end of each visit gave the expectant parents an opportunity to get acquainted with the birth rooms, which the guides believed further strengthened parents’ feeling of safety. The women also shared birth experiences from their home countries and the conversation could become both personal and in-depth:

*‘Yes, sometimes it comes up, that maybe the first birth was horrible, or that they were very afraid of certain things, that they didn't receive any pain relief. And then I explain how the Swedish healthcare system works and a Swedish delivery with pain relief and how it is, and stuff.'* (Guide 1)

*Being genuinely engaged with each participant*

The guides described INFOR being about building a bridge between the woman and the guide, primarily on a personal level. Trust and security can be created through mutual exchange without judgement, being genuinely present in the conversation and listening to expectant parents' individual needs and concerns, where all parties are interested in understanding each other. The guides thought trust and security reduced the risk of complications during childbirth. The visits required the ability to think ‘outside the box' and the usual professional role. Being interested in the unique person and wanting to work with contexts other than the traditional Swedish context were raised as cornerstones for success.

The guides found it essential to have a genuine interest in migrants and to understand their need for extra support, given their lack of familiarity with the Swedish healthcare system. They expressed the view that equal care does not mean that everyone should be given the same support and information, but rather be given care based on the needs of the individ person:

*‘It's about cultural ability and knowledge, it takes time. But most of all, like, we have to think outside our ordinary boxes, as Swedish healthcare professionals. It's about cultural knowledge.’* (Guide 6)

The ability to be personal, responsive, flexible, and unselfish and to work with one's heart determined whether the guides felt that they could connect to the woman or not. According to the guides, being passionate about the work, helping each other and having the will to do what it takes were all crucial elements in improving the expectant parents' chances of a positive birth experience:

*'... if I notice that, oh, they could really do with coming in one more time, then I offer them that. I have done so, maybe in fact, half of the times, … if they want to come again. Sometimes they don't want that, but sometimes they do ... Or sometimes it feels just obvious that now, now it's done. So, now they are ready to … now they feel a little safer and it feels a little better.'* (Guide 3)

*Challenges faced*

The guides had a desire to give all pregnant women person-centered and equitable care, but in reaching that goal they mentioned barriers too. The guides also described how they experienced the work with INFOR on an emotional and personal level. Three subthemes describe these challenges: *Communication via an interpreter, Adapting the visits to the individual and their different prerequisites and Developing and enriching, but not sufficient.*

*Communicating via an interpreter*

According to the guides, the interpreters had a significant impact on how the visits turned out. The guides described considerable variation in the quality of interpreting where a skilled interpreter made the visit better and contributed to the woman’s sense of security. A female interpreter with healthcare competence was seen as particularly beneficial:

*'… a good example … one doesn't notice that there is another person in the room. I and the woman have the dialogue eye to eye, or I and the partner and the woman, so that we don't have a person who takes attention from the conversation, where I also notice that a feeling of safety is created in the room, so that I can trust that this will be a good translation, this is done correctly. There won't be a lot of extra words, it won't be a lot of silence but what I hear, what I inform about and what I listen to, sounds roughly the same ...'* (Guide 2)

Communication via an interpreter requires a lot from the guide and opinions differed about whether a face-to-face or telephone interpreter is preferable. Face-to-face interpreting strengthened the communication through non-verbal cues and rapport building, but the presence of the interpreter could sometimes be too dominant in the communication, and guides reported that sometimes there was a tendency towards not interpreting verbatim, but possibly adding one’s own values and interpretation to what was said. One advantage of telephone interpreting was that it was less personal and that anonymity could be maintained. A disadvantage of telephone interpreting, however, was that the conversation could become superficial and less comprehensive:

*'It is very (interpreter over phone) … I think it is not so much help. It's better than nothing but to get it right … Help, with how everything is … Better with a direct contact but of course, this is not always possible.'* (Guide 4)

One way to improve the communication was to prepare the interpreter, to make her/his role clear right from the start. The guides reported that they sometimes had to break in and question the interpreter’s interpretation when shortcomings were experienced. Further, on some occasions the guides felt a need to explain to the interpreter that the translation should be done verbatim.

*Adapting the visits to the unique individuals and their different prerequisites*

The guides described the visits as needing to be adapted according to the expectant parents’ knowledge and needs. They described migrant women as a vulnerable group with increased risks of obstetric complications and that they should have access to additional support and resources because of this, which they did not always receive. The guides were motivated to help women with everything they needed, and the families were reported to be often very grateful for the help they received during the visits. However, it was not always possible to help with everything, which felt frustrating. Some of the visits led to referral of the woman for birth planning, or for more support, due to their fear of childbirth. Ensuring the same preconditions before birth for migrant women as for Swedish-born women, was seen an important task by the guides: the same for all, creating equality, as pointed out in the Swedish Healthcare Act. In addition, the guides said that they wanted to give the INFOR-families more than the standard maternity care:

*'… I'm a total chameleon, I would probably say. Yes. Which is in a way, abandoning the Swedish standard care model.’* (Guide 6)

The guides pointed out that many of the women they had met came to them with difficult histories, including trauma or violence during pregnancy, experience of genital mutilation, sex-related violence, past negative birth experiences, and having lived as refugees. Most guides described the importance of finding out about such previous experiences to connect with each woman, create trust and make her feel safe so that she would have the best chance of a good birth experience. The guides also said that establishing personal contact during a visit could be difficult as conversations about certain issues could be sensitive and stigmatizing, which sometimes created distance. Daring to ask questions was difficult and the guides were not always able to dig as deep into the conversation as they wished, even though they had a feeling that the information was important for reducing risks in connection to pregnancy, birth and postpartum.

*Developing and enriching but not sufficient*

The guides described the visits as often being emotional and personal, and that the women and their concerns stayed with them long after the sessions. The desire to provide more help than could be covered during the visit could awaken feelings of inadequacy. However, being able to help women in difficult situations and make them feel seen and valued was described as enriching, positive and rewarding by the guides. All guides felt that INFOR made a difference and had a positive impact on the participating families. The guides felt that the women were grateful after the visit, that they also looked happier and seemed calmer. When the guides had asked what the women liked about the visits, they replied that they felt satisfied and more confident about the upcoming birth:

*‘I haven't had a single woman or man leaving the visit unaffected.'* (Guide 1)

The guides stated that they often noticed a difference when women arrived at the labor ward to give birth, between the non-Swedish-speaking migrants who had participated in INFOR and those who had not. The guides wanted to be able to demonstrate that the INFOR visits had positive outcomes, but they acknowledged the difficulty of measuring this quantitatively:

*'They are quite tense when they arrive with us, and so, they are very, you can see, they are very closed in the beginning. Then you notice, at the end it is like “wow", it will be so much fun to read her evaluation later on. You understand them so quickly, they are so skeptical and hesitant about it all “what is this?" Then you see how they, like, become more and more, they loosen up and feel that this, like, that it feels good.'* (Guide 5)

### Wanting to sustain and develop the model

This theme describes the guides’ thoughts and hopes for the future of INFOR, hopes that matched the original ambitions for INFOR. The two subthemes are: Content and structure of the individual visit and Reaching out to more parents in need of INFOR.

*Content and structure of the individual visit*

The importance of individualized and person-centered care was expressed by the guides, but in order to build a relationship to increase the women’s feelings of safety, they felt that there was a need for repeated contact. Some of the guides felt that the scheduled time for the individual visit was too short and that it took place too late in pregnancy. Better cooperation with community antenatal care midwives, but also with social authorities, came up as suggestions for improvement. The guides wanted to make INFOR more visible and felt a desire to set up a clear goal for the project:

*'…but we have to like, set up goals that are connected to that, clinically speaking that is. For example, we have said that it would be very exciting to be Sweden's first migrant friendly hospital.'* (Guide 2)

*Reaching out to more parents in need of INFOR*

The guides felt that INFOR did not reach everyone in need of the visits. They mentioned different ways to reach more women/families and how important it is to think creatively to achieve this. They emphasized greater engagement with community antenatal care services for recruiting women and their families. Inability to communicate the purpose of INFOR to the women directly was also raised as problematic. The guides felt that some of the women and their families did not understand initially why they should come to INFOR, something which could have led to missed visits. However, the guides believed that the families who were hesitant in the beginning, not really understanding the purpose, appeared to be happy once they arrived and met the guide.

## DISCUSSION

The experience of providing extra support to migrant pregnant women through INFOR was described by the guides as being a bridge and creating safety, which was achieved by meeting with women and providing practical information. The guides felt that they fulfilled an important purpose, they were dedicated and adaptable to meet the women’s individual needs, but they highlighted some barriers. Communicating via an interpreter was a challenge and the women were often in need of more and extended visits. The guides wished that INFOR could become a standard part of antenatal care but felt that the model needed further development and a better system for recruitment of women.

Consistent with our findings in INFOR, maternity service health professionals working with refugee families in Australia, recognized that acknowledging refugee women’s migration history, being friendly, showing interest in their lives and having enough time for appointments were important for being able to provide high quality care^23^. Refugees interviewed in the same study also described how these factors impacted on their care experiences.

The INFOR-guides described trying to create safety for the INFOR-participants by engaging with each participant, acknowledging and learning about their history, and giving them relevant information. As findings from another Swedish study show, undocumented migrants in Sweden felt acknowledged and empowerment when meeting healthcare professionals who actively made an effort to establish a trusting relationship^24^. When the women felt welcome, were treated respectfully and equal to others, they felt safer. Additionally, the women reported an increased level of trust when the healthcare professionals were available to answer questions outside the standard antenatal care visits, very much like the opportunity provided women in INFOR. In a recent systematic review exploring migrant women’s experiences of pregnancy, childbirth and maternity care, four overarching themes were outlined^25^. All the issues raised by the women in the review, were issues the guides in INFOR aimed to address: *1) Finding the way—navigating the system in a new place, 2) We don't understand each other, 3) The way you treat me matters, and 4) My needs go beyond being pregnant.*

Pregnant women who have recently migrated to another country and who do not speak the national language comprise a vulnerable group in need of extra support, which is one of the reasons that INFOR was started. Fearing the unknown is one of the most common reasons for fear of childbirth^26^ and earlier research has shown that fear of birth is more prevalent among foreign born women^27^. When physical and psychosocial health problems were investigated among women migrating to Australia, they had more anxiety in early pregnancy, compared to Australian-born women^28^. Seeing a healthcare professional working at the hospital during pregnancy with communication support in place, as happened in INFOR, might have a positive impact on migrant women’s psychosocial health and mean that participating women have fewer fears connected to birth after such a visit. The migrant women who participated in INFOR reported positive experiences of the visits in interviews conducted for the evaluation of INFOR (Ternström E, et al. unpublished data, 2021), confirming the guides’ perceptions of the benefits.

When interviewing midwives about caring for immigrant women in the Netherlands and Denmark, low educational level, health literacy and lack of knowledge about the maternity care system were expressed as difficulties^15,29^. The women expected the same care as they were used to in their country of origin and the midwives had to spend extra time explaining the process in the new country, also documented in another recent study from Sweden^12^. This created dissatisfaction and frustration for both midwives and women. If community antenatal care midwives know about the INFOR model, the hospital and community sectors can cooperate productively in women’s interests, and if the guides have enough time with the expectant parents during the visit to labor ward, the model may be a good way to prevent such frustration and dissatisfaction. The guides in INFOR expressed a need for more visits and one way forward could be to have a first visit at the beginning of pregnancy.

The challenges involved in working with interpreters and the differing skill level of interpreters were described by the guides in this study, and similarly also by midwives in Ireland caring for asylum-seeking women^30^. According to midwives in Sweden in another study, communication problems play a central role in health inequalities for migrants and education is a more important factor than culture in relation to women’s use of healthcare^13^. A complement to INFOR for improving care for migrants could be to develop resources, similar to the ongoing Danish MAMAACT intervention^15^, which includes a leaflet and an app in different languages, alongside pictures and audio messages for women with low levels of literacy, as a means of addressing ethnic disparities in mother and child health. Providing cultural responsiveness training and ensuring all guides have had training in working with interpreters may be ways forward in developing increased safety and trust for women in INFOR and enhanced support for the guides. Midwives’ knowledge, skills and cultural competence significantly increased after culturally sensitive maternity care training shown in the ORAMMA project^31^.

The guides in INFOR described how the visits with migrant families were evolving over time and they felt that they fulfilled an important purpose. However, they reported that women often told stories about traumatic experiences and the guides frequently wanted to provide more help than they could offer. Similarly, midwives in the Netherlands described caring for migrants as demanding but rewarding^29^ and other studies show that midwives felt ill-equipped to handle all the stories that emerged^12,30^. Moreover, the INFOR-guides expressed a need for reflection and guidance and a future development for INFOR could be to involve a counsellor in the project, as a support for both guides and expectant families.

All guides wanted to continue caring for migrant women and their families in INFOR and had a desire to extend it to other clinics. They saw the positive effects of the visits in creating safety for the women. The Public Health Agency of Sweden recently published the evidence-based report ‘Health of people born abroad - differences in health based on country of birth’ in which it outlined the importance of reducing health inequalities^32^. The report describes the importance of newly arrived migrants receiving information about the healthcare system and that support structures need to be established. High quality care should be given to all, regardless of background, and person-centered care adapted to the individual needs is described in the report as critical to achieving quality care provision.

### Methodological considerations

Thematic analysis is a flexible method which enables different analytic approaches. The credibility of the study was enhanced by the heterogeneity in participant age and level of experience. Further, the interviews took place when the INFOR project was still ongoing, reducing recall bias. The guides were interviewed face-to-face which is advantageous for non-verbal communication and rapport building. The interviews were recorded and transcribed, and data were analyzed by going back and forth to interpret the interviews. Transferability and dependability were enhanced through the detailed description of the context, project, data collection, analysis and findings. Further, the interviews provided rich and in-depth descriptions of the guides’ experiences which should ensure enough information for assessment by other researchers. Additionally, the analysis was performed step by step, reading the data back and forth, by several researchers, individually and together. Conformability was assured as the people performing the interviews were not involved in implementing INFOR, which is advantageous for objectivity and neutrality. When analyzing the data, researchers’ own beliefs and position regarding the research area can affect the results and the conclusions. However, the checklist used for undertaking sound thematic analysis and the comprehensive analysis process including agreement between five authors, probably diminishes the risk for subjective bias.

A shortcoming in the study may be that anonymity is difficult to maintain as only eight guides worked with INFOR at the time, and six of them participated in the interviews. Further, no information is available on why two guides declined to be interviewed. The findings might have been different if the reason for declining was due to an experience of INFOR that differed from the guides who did participate. However, there is little reason to believe that this was a major problem, as the guides who were interviewed described all the guides as having positive experiences overall. The guides were both assistant nurses and midwives and it is possible that the content of the visits with the women and their families varied due to differing training and roles, but this should not have been a major problem as visits were intended to be responsive to women’s needs, so all guides were led by women themselves in terms of the content covered.

## CONCLUSIONS

Migrant women giving birth in Sweden comprise a group often needing extra support. Providing individual language-supported hospital visits with healthcare professionals may assist in enhancing communication and support and thus achieve more equitable maternity care. The participating guides believed that the INFOR model was a valuable adjunct to standard antenatal care for migrant women. They valued the opportunity to inform and support pregnant migrant women and believed that the expectant couples greatly appreciated the model.

For future implementation of the INFOR model, it would be valuable to articulate specific goals and a structured protocol to follow to maximize the opportunity to achieve equitable care. In addition, however, the guides should also have room to broaden the focus of the visit, depending on the women’s and their families’ needs. To achieve optimal communication with the expectant parents, participating healthcare professionals should have adequate training in working with interpreters. Improving the recruitment processes for INFOR should also be a priority, if all migrant women in need are to be offered INFOR in future. This might require the development of more formal collaboration and protocols between the hospital and community-based antenatal care midwives and social authorities. The findings here suggest that the INFOR model might usefully be implemented in other hospital labor ward settings to improve the care of migrant women, with evaluation of pregnancy outcomes and women’s experiences an essential part of further implementation and research.

## Data Availability

The data supporting this research are available from the authors on reasonable request.
